# Raloxifene inhibits adipose tissue inflammation and adipogenesis through Wnt regulation in ovariectomized rats and 3 T3-L1 cells

**DOI:** 10.1186/s12929-019-0556-3

**Published:** 2019-08-31

**Authors:** Hsin-Hsueh Shen, Chien-Yi Yang, Ching-Wen Kung, Shu-Ying Chen, Hong-Min Wu, Pao-Yun Cheng, Kwok-Keung Lam, Yen-Mei Lee

**Affiliations:** 1 Department and Institute of Pharmacology, National Defense Medical Center, Taipei, Taiwan; 2 Graduate Institute of Medical Sciences, National Defense Medical Center, Taipei, Taiwan; 3 Department of Pharmacy Practice, Tri-Service General Hospital, National Defense Medical Center, Taipei, Taiwan; 4 Division of General Surgery, Department of Surgery, Tri-Service General Hospital Sungshan Branch, Taipei, Taiwan; 5 Department of Nursing, Tzu Chi University of Science and Technology, Hualien, Taiwan; 6 Department of Nursing, Hung Kuang University, Taichung, Taiwan; 7 Department of Physiology & Biophysics, National Defense Medical Center, Taipei, Taiwan; 8 Department of Pharmacology, Taipei Medical University, Taipei, Taiwan; 9 Department of Anesthesiology, Catholic Mercy Hospital, Hsinchu, Taiwan

**Keywords:** Raloxifene, Menopause, Obesity, White adipose tissue, Inflammation, Wnt10b, β-Catenin, Wnt5a, SFRP5

## Abstract

**Background:**

Loss of ovarian function, as in menopause or after ovariectomy (OVX), is closely associated with obesity and white adipose tissue (WAT) inflammation. Estrogen replacement protects against postmenopausal obesity but increases the risks of carcinogenesis. In the present study, we investigated the effects of long-term treatment of raloxifene (RAL), a selective estrogen receptor modulator, on the features of estrogen deficiency-induced obesity and explored the involvement of canonical and non-canonical Wnt regulation in vivo and in vitro.

**Methods:**

Adult female rats received bilateral OVX and divided into 5 groups: (1) Sham, (2) OVX, (3) OVX + E_2_: OVX rats were administered with E_2_ (50 μg/kg, s.c., 3 times/week), (4) OVX + RAL: OVX rats were treated with RAL (gavage, 1 mg/kg/day) suspended in 0.8% carboxymethylcellulose (CMC), (5) OVX + CMC: 0.8% CMC as vehicle control. All treatments were given for 8 weeks beginning at 1 week after OVX. In 3 T3-L1 cells, the effects of RAL on adipogenesis and lipopolysaccharide (LPS)-induced inflammation were evaluated.

**Results:**

Treatment with RAL significantly decreased body weight, visceral fat pad mass, adipocyte size and plasma levels of glucose but increased plasma adiponectin. RAL reduced the elevation of HIF-1α, VEGF-A and proinflammatory cytokines (MCP-1 and TNF-α) expression by inhibition of NF-κB p65 and JNK cascades in retroperitoneal WAT. This anti-inflammatory capacity of RAL may result from upregulation of secreted frizzle-related protein 5 (SFRP5), an adipokine that repressed Wnt5a signaling. Furthermore, RAL inhibited adipogenic factors such as PPAR-γ, C/EBP-α, and FABP4, and preserved canonical Wnt10b/β-catenin protein expression. In 3 T3-L1 adipocytes, RAL (20 μM) diminished lipid accumulation and inhibited adipogenic factors accompanied with the induction of β-catenin, which were effectively reversed by the β-catenin inhibitor IWR-1-endo. In addition, RAL reduced LPS-induced NF-κB p65 and p-IκB expression as well as TNF-α secretion. Suppression of SFRP5 by small interfering RNA significantly abrogated the anti-inflammatory effects of RAL.

**Conclusions:**

Distinct activation of canonical β-catenin on inhibition of adipogenesis and non-canonical SFRP5 on suppression of WAT inflammation may contribute to the beneficial effects of RAL. Therefore, this study provides a rationale for the therapeutic potential of RAL for postmenopausal obesity.

**Electronic supplementary material:**

The online version of this article (10.1186/s12929-019-0556-3) contains supplementary material, which is available to authorized users.

## Introduction

Postmenopausal women have a higher risk of developing obesity and metabolic syndrome caused by loss of ovarian function than premenopausal women [1]. Increasing evidence supports that estrogen not only modulates the reproductive function but also plays a critical role in the regulation of adipocyte differentiation, lipid metabolism and insulin sensitivity [2, 3]. Moreover, decreased systemic levels of estrogen after menopause or ovariectomy (OVX) resulted in the progression of visceral fat accumulation and chronic low-grade inflammation in adipose tissue [4]. Thus, understanding the contribution of estrogen deficiency in the pathogenesis of metabolic syndrome is emerging as a new therapeutic concern.

White adipose tissue (WAT) is an important depot for energy storage and endocrine organ for the secretion of adipokines, e.g., leptin and adiponectin. These adipokines play an important role in the regulation of glucose homeostasis and insulin sensitivity [5]. Excessive accumulation of visceral WAT in obesity causes adipocyte hypertrophy and hypoxia [6], leading to the induction of the key hypoxia transcription factor, hypoxia-inducible factor-1α (HIF-1α) and substantial secretion of pro-inflammatory chemokines, including monocyte chemotactic protein-1 (MCP-1) [7] and tumor necrosis factor-α (TNF-α) [8]. Overt secretion of these adipokines may contribute to the pathogenesis of systemic insulin resistance and type 2 diabetes.

Wingless-type MMTV integration site family members (Wnts) are a family of secreted glycoproteins that possess extensive autocrine and paracrine effects on cellular differentiation and growth [9]. Accumulating evidence has prompted that Wnt signaling not only regulates adipogenesis [10] but also inflammatory responses of WAT [11]. Adipogenesis is mainly regulated by various adipogenic transcriptional factors, such as peroxisome proliferator-activated receptor-γ (PPAR-γ), and CCAAT/enhancer-binding protein α (C/EBP-α) [12]. The canonical Wnt/β-catenin pathway acts as a negative regulator of adipogenesis by downregulation of these adipogenic transcriptional factors [13]. In the absence of Wnt activation, β-catenin is rapidly degraded by glycogen synthase kinase (GSK) 3β-AXIN-adenomatous polyposis coli (APC) complex [14]. When Wnt ligand, such as Wnt10b, binds to Frizzled (Fz) receptors and low-density lipoprotein receptor-related protein (LRP) coreceptors, disheveled (DVL) s are activated to enhance the disruption of GSK3β-AXIN-APC complex and stabilize β-catenin. This stabilization increases translocation of β-catenin into nucleus and represses the synthesis of adipogenic transcription factors [13]. On the other hand, Wnt5a is classified as a non-canonical Wnt protein, which secreted by infiltrated macrophages in the WAT and activates β-catenin-independent signaling to augment inflammatory responses [15]. These inflammatory responses enhanced Wnt5a to activate c-Jun N-terminal kinase (JNK) cascade and resulted in the progression of insulin resistance [16]. In addition, the secreted frizzled-related protein 5 (SFRP5) sequesters Wnt5a in the extracellular space and hinders it from binding to the associated receptors. It is well established that SFRP5 is expressed in the WAT of lean mice but downregulated in extremely obese mice [17], and has been emerged as an anti-inflammatory adipokine to protect against insulin resistance [18]. Thus, manipulation the balance between Wnt5a and SFRP5 may represent a potential strategy for management of obesity-associated metabolic abnormalities.

Selective estrogen receptor modulators (SERMs) are developed as an alternative postmenopausal hormone therapy with less incidences of endometrial and breast carcinogenesis through acting as estrogen receptor agonists or antagonists depending on the distinct tissues. Raloxifene (RAL) is the second generation of SERMs clinically approved for the prevention of osteoporosis during menopause, which exerts anti-estrogenic activity in breast and estrogenic activity in bone [19]. Additionally, RAL was reported to reduce circulating MCP-1 and enhance peripheral insulin sensitivity in post-menopausal women [20]. In rodents with OVX, RAL prevented weight gain and fat pad mass by modulating hyperleptinemia and attenuated serum cholesterol in a dose-dependent manner [21, 22]. Although RAL is evident to improve metabolic dysfunctions during estrogen deficiency, its underlying mechanism remains to be elucidated. The present study aimed to investigate the beneficial effects of long-term treatment with RAL against estrogen deficiency-induced obesity and metabolic dysfunctions in OVX rats and further explored the role of Wnt signaling using 3 T3-L1 adipocytes.

## Materials and methods

### Animal preparation

Female Wistar rats (7 weeks old, 250–270 g) were purchased from BioLASCO Taiwan Co., Ltd., Taiwan. Animal Handling was in accordance with the Guide for the Care and Use of Laboratory Animals published by the US National Institutes of Health (NIH Publication No. 85–23, revised in 1996). This study was approved by the Institutional Animal Care and Use Committee of the National Defense Medical Center, Taiwan (Number of Permission: IACUC-11-116). To generate estrogen-deficiency condition, rats were anesthetized with sodium pentobarbital (50 mg/kg, intraperitoneal injection [i.p.]) and subjected to bilateral ovariectomy (OVX) at 8 weeks old, as previously described [23].

### Experimental groups

One week after OVX, rats were divided into five groups: (I) Sham: rats were anesthetized and subjected to sham operations (*n* = 8); (II) OVX: rats were ovariectomized bilaterally (*n* = 10); (III) OVX+ E_2_: OVX rats were administered with 17β-estradiol (E_2,_ 50 μg/kg, s.c., three times a week) (Sigma-Aldrich, St. Louis, MO, USA) for 8 weeks beginning at 1 week after OVX (to deplete endogenous sex hormones) (*n* = 10); (IV) OVX + RAL: OVX rats were administrated with RAL (gavage, 1 mg/kg/day) (Sigma-Aldrich) for 8 weeks beginning at 1 week after OVX. RAL was suspended homogeneously in 0.8% carboxymethylcellulose (CMC, Sigma-Aldrich) (*n* = 9); (V) OVX + CMC: OVX rats were administrated with 0.8% CMC (gavage, 1 ml/kg/day) for 8 weeks beginning at 1 week after OVX (*n* = 8). After 8 weeks of treatment, rats were then sacrificed to collect blood and adipose tissue samples for further analysis.

### Measurement of plasma levels of E_2_, adiponectin and glucose

Blood samples (1 mL) were withdrawn by cardiac puncture and centrifuged at 12,000 *g* for 5 min at 4 °C. The supernatants of the blood samples were collected and subjected to the following measurements. Plasma levels of E_2_ were determined by luminescence immunoassay (Automated Chemiluminescence System, Bayer, Co. NY, USA); Plasma levels of adiponectin were measured using enzyme-linked immunosorbent assay kit (Abcam, Cambridge, MA, USA); Plasma glucose levels were detected by a One Touch II blood glucose monitoring system (Lifescan, Milpitas, CA, USA).

### Hematoxylin & Eosin stain

Retroperitoneal WAT was fixed using 10% paraformaldehyde diluted in PBS, embedded in paraffin, and then sectioned for histological analysis. Hematoxylin and Eosin (HE) stain was performed according to the standard procedure. Digital images of HE-stained tissue sections were performed for adipocyte size analysis with ImageJ Program. The average adipocyte size was expressed as the average cross-sectional area per cell (μm^2^/cell) of tissue sample and calculated based on the values of at least 20 adipocytes.

### Western blot analysis

Total proteins were extracted using RIPA lysis buffer within 1% protease inhibitor cocktail (Millipore, Bedford, MA, USA) 0.1 mM according to the manufacturer’s instructions. Protein samples were separated using 10% sodium dodecyl sulfate-polyacrylamide gels and transferred to a nitrocellulose membrane (Millipore, Bedford, MA, USA). After blocking, the membranes were then incubated at 4 °C overnight with the following primary antibodies: anti-HIF-1α, anti-VEGF-A, anti-JNK, anti-PPAR-γ, anti-C/EBP-α, anti-FABP4 (1:1000, all Cell Signaling Technology, Danvers, MA, USA), anti-NF-κB p65, anti-p-IκB, anti-TNF-α, anti-MCP-1 (1:1000, all Abcam, Cambridge, MA, USA), anti-SFRP5, anti-Wnt5a, anti-Wnt10b, anti-β-catenin (1:1000), and anti-β-actin (1:5000, all GeneTex, Irvine, CA, USA). After washing, the membranes were probed with corresponding second antibodies (1:3000, GeneTex). The density of the individual protein bands was quantified by densitometric scanning of the blots using ImageJ software.

### Cell culture and differentiation

Mouse 3 T3-L1 fibroblasts (American Type Culture Collection, ATCC, Manassas, VA, USA) were cultured in DMEM with 10% BCS at 37 °C in 5% CO_2_ atmosphere until the confluence of the cells. Differentiation was induced 2 days post-confluence (differentiation day 0) by replacing the medium to DMEM with 10% FBS (not charcoal stripping) plus MDI, including 0.5 mM IBMX, 1 μM dexamethasone, and 10 μg/mL insulin. After incubation for 3 days, the culture medium was replaced with fresh DMEM containing 10% FBS and insulin (10 μg/mL) every 3 days. The cells were fully differentiated into mature adipocytes on Day 9. During the differentiation process (Day 0 to 9), cells were treated with various concentrations of RAL (1–20 μM), according the schematic protocol (Fig. 7b). The passages of 3 T3-L1 cells used in these experiments were 6–12.

### Cell viability

The 3 T3-L1 cells were treated with RAL (1–20 μM) in 96-well plates for 16 h. Cell viability was assessed using a 3-(4,5-dimethylthiazol-2-yl)-5 -(3-carboxymethoxyphenyl)-2-(4-sulphophenyl)-2H-tetrazolium (MTS)-based CellTiter96® AQueous One solution kit (Promega, Madison, WI, USA), according to the manufacturer’s directions.

### Oil red O staining

At the end of differentiation, cells were stained by Oil Red O (Sigma-Aldrich) for determination of lipid accumulation and visualized using bright-field microscopy.

### Transfection with small interfering (si) RNA for SFRP5 knockdown and LPS treatment in 3 T3-L1 cells

Two days after confluence, 3 T3-L1 cells were transfected with a non-targeting control or SFRP5 siRNA (final concentration 25 nM; Dharmacon, Lafayette, CO, USA) according to the manufacturer’s instructions. After 24 h, the transfected cells were differentiated following the differentiation protocol. Lipopolysaccharide (LPS) 10 μg/mL (LPS, Sigma, St Louis, MO, USA) and RAL (20 μM) were treated on Day 8 and following the differentiation process until Day 9.

### Enzyme linked immunosorbent assay (ELISA)

LPS-treated cultured medium was collected for the measurement of TNF-α levels using ELISA kit (Cloud-Clone Corp., Houston, USA), following the manufacture’s instruction.

### Statistical analysis

The data are expressed as means± SEM. A *Two-Way ANOVA and* LSD post-hoc comparison test *was used to* compare the means of body weight between the groups and time. Statistical evaluation of others was performed with one-way analysis of variance (ANOVA) followed by the Newman–Keuls method. A value of *P* less than 0.05 was accepted as indicating statistical significance.

## Results

### Effects of RAL on plasma levels of E_2_, body weight (BW) and total visceral fat pad mass in OVX rats

**Fig. 1:**
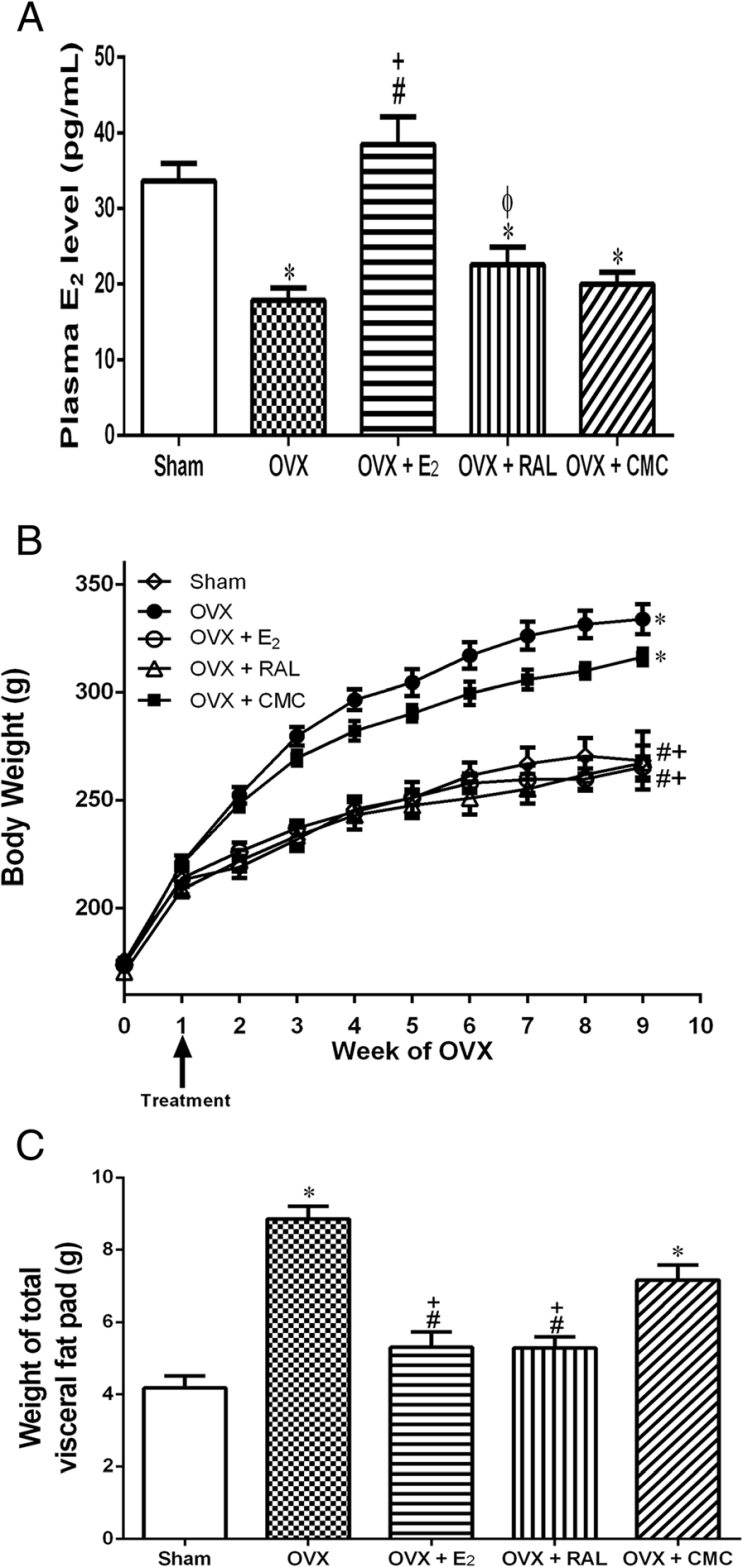
Effects of long-term treatment with raloxifene on ovariectomy-induced obesity in rats. **a** Plasma E_2_ level, (**b**) time-course changes of body weight, and (**c**) total visceral fat pad mass were measured after raloxifene treatment for 8 weeks. Data are expressed as mean ± SEM. *P < 0.05 versus Sham; ^#^ *P* < 0.05 versus OVX; ^+^*P* < 0.05 versus OVX + CMC; ^ϕ^*P* < 0.05 versus OVX + E_2_, *n* = 8–10. OVX: ovariectomy; RAL: raloxifene (1 mg/kg/day, gavage); E_2_ (50 μg/kg, s.c.); CMC: carboxymethylcellulose (1 ml/kg/day, gavage)

Nine weeks after OVX, the plasma levels of E_2_ in the OVX (17.9 ± 1.5 pg/mL), OVX + RAL (22.6 ± 2.3 pg/mL), and OVX + CMC groups (19.9 ± 1.6 pg/mL) were significantly reduced as compared with the Sham group (33.7 ± 2.3 pg/mL). Replacement of E_2_ significantly reversed the decreased levels of E_2_ (38.5 ± 3.6 pg/mL) (Fig. 1a).

Nine weeks after OVX, the BW of OVX (333.8 ± 6.9 g) and OVX + CMC (316.3 ± 3.8 g) groups were significantly increased as compared to the Sham group (268 ± 13.4 g) (*P* < 0.05). The OVX + E_2_ (265.4 ± 4.9 g) and OVX + RAL (267.3 ± 3.5 g) groups showed significant lower levels of BW than those of OVX and OVX + CMC groups (Fig. 1b).

Total visceral fat pad mass was consisted of peri-renal, retroperitoneal and mesentery fat pad mass. Figure 1c showed OVX (8.8 ± 0.3 g) and OVX + CMC (7.2 ± 0.4 g) groups exhibited significant increases in total visceral fat pad mass when compared with the Sham group (4.1 ± 0.3 g). The increase of fat pad mass was significantly reduced in OVX + E_2_ (5.3 ± 0.4 g) and OVX + RAL (5.2 ± 0.3 g) groups as compared with OVX and OVX + CMC groups (*P* < 0.05).

### Effects of RAL on plasma levels of adiponectin and glucose in OVX rats

**Fig. 2:**
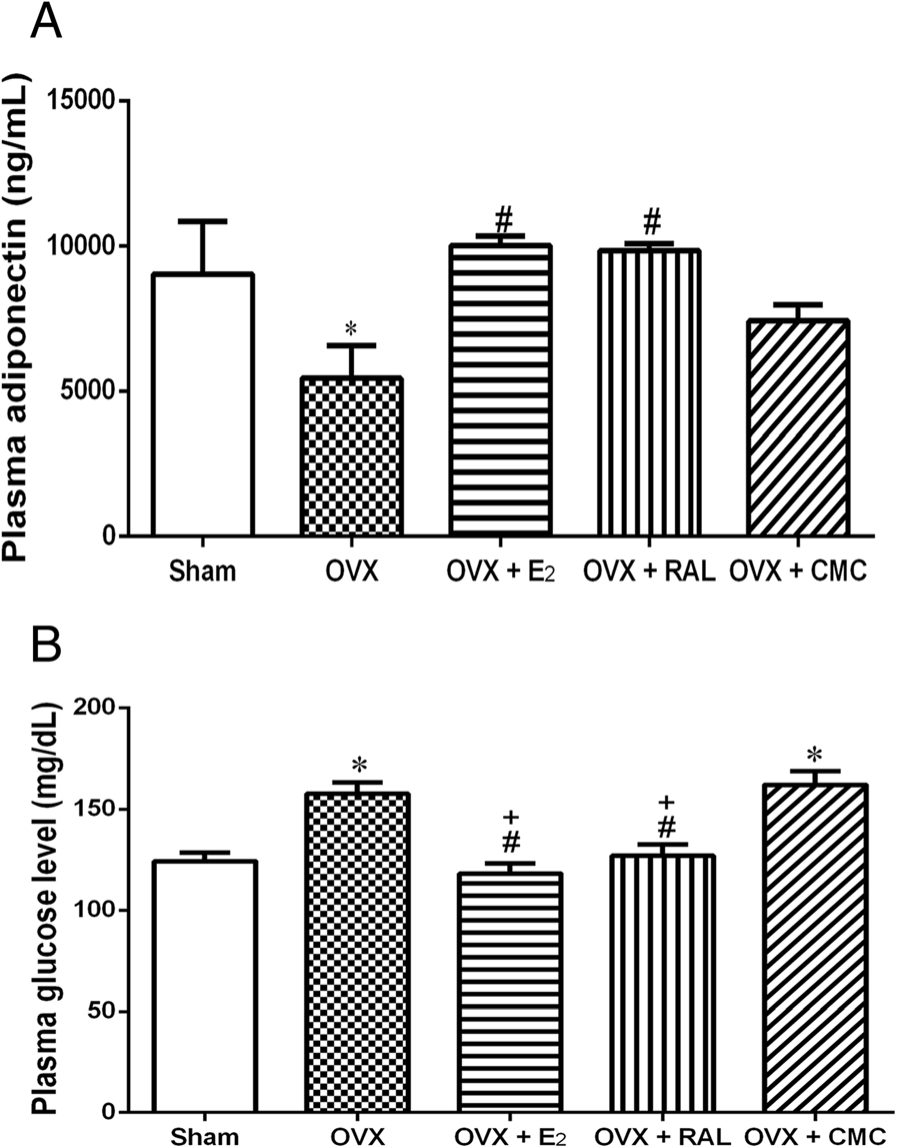
Effects of long-term treatment with raloxifene on metabolic parameters in ovariectomized rats. **a** Plasma adiponectin and (**b**) glucose levels were measured after raloxifene treatment for 8 weeks. Data are expressed as mean ± SEM. *P < 0.05 versus Sham; ^#^ *P* < 0.05 versus OVX; ^+^*P* < 0.05 versus OVX + CMC, *n* = 8–10. OVX: ovariectomy; RAL: raloxifene (1 mg/kg/day, gavage); E_2_ (50 μg/kg, s.c.); CMC: carboxymethylcellulose (1 ml/kg/day, gavage)

As shown in Fig. 2a, plasma levels of adiponectin were significantly decreased in the OVX group as compared with the Sham group, however, both replacement of E_2_ and long-term treatment with RAL markedly elevated plasma adiponectin levels (*P* < 0.05). In addition, OVX induced a significant increase in plasma levels of glucose when compared with the Sham group, which were significantly attenuated by E_2_ and RAL treatment (*P* < 0.05; Fig. 2b).

### Effects of RAL on adipocyte size in OVX rats

**Fig. 3:**
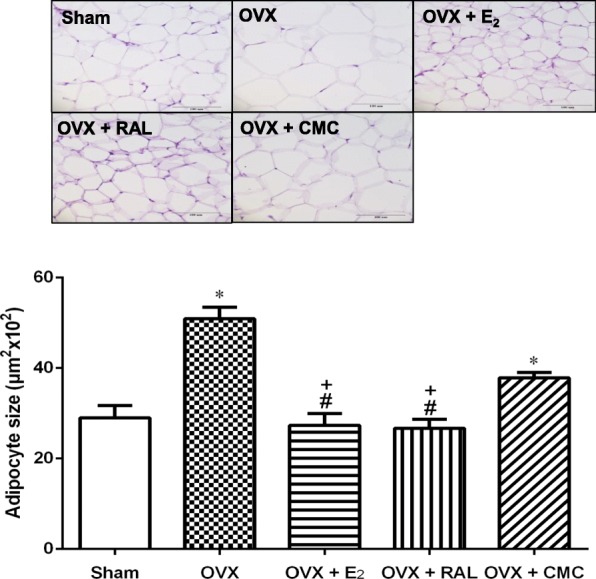
Effects of long-term treatment with raloxifene on adipocyte size of retroperitoneal white adipose tissue in ovariectomized rats. Representative photomicrographs of cross sections after HE staining. Data are expressed as mean ± SEM. *P < 0.05 versus Sham; ^#^ *P* < 0.05 versus OVX; ^+^*P* < 0.05 versus OVX + CMC, *n* = 8–10. OVX: ovariectomy; RAL: raloxifene (1 mg/kg/day, gavage); E_2_ (50 μg/kg, s.c.); CMC: carboxymethylcellulose (1 ml/kg/day, gavage). The images were captured at 200× magnification

Adipocyte size of retroperitoneal WAT was assessed using HE stain to observe whether adipocyte hypertrophy occurred after OVX. As shown in Fig. 3a, the adipocyte size in OVX (59.2 ± 2.6 × 10^2^ μm^2^) and OVX + CMC groups (37.9 ± 1.2 × 10^2^ μm^2^) was significantly higher than that of the Sham group (29.0 ± 2.7 × 10^2^ μm^2^). Both administration of E_2_ (27.4 ± 2.6 × 10^2^ μm^2^) and RAL (26.7 ± 2.0 × 10^2^ μm^2^) in OVX rats significantly reduced the adipocyte size (*P* < 0.05).

### Effects of RAL on adipocyte hypertrophy-induced inflammatory responses in OVX rats

**Fig. 4:**
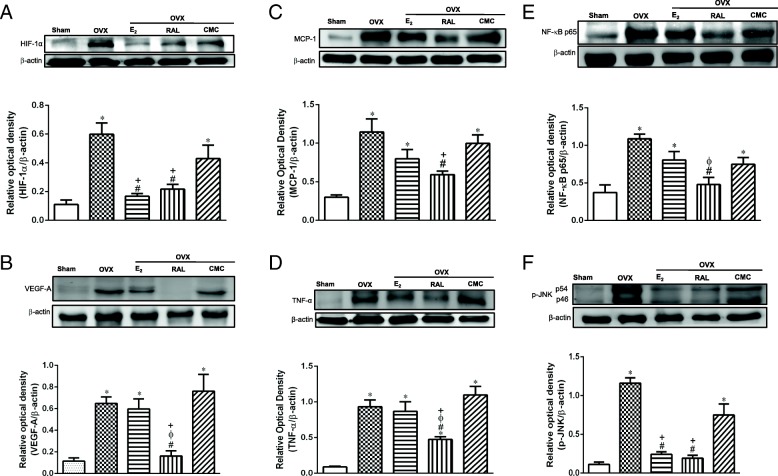
Effects of long-term treatment with raloxifene on hypoxia and inflammatory responses in the retroperitoneal white adipose tissue in ovariectomized rats. Expression levels of (**a**) HIF-1α, (**b**) VEGF-A, (**c**) MCP-1, (**d**) TNF-α, (**e**) NF-κB p65, and (**f**) p-JNK in retroperitoneal white adipose tissue. Data are expressed as mean ± SEM. *P < 0.05 versus Sham; ^#^ *P* < 0.05 versus OVX; ^ϕ^*P* < 0.05 versus OVX + E_2_; ^+^*P* < 0.05 versus OVX + CMC; ^ϕ^*P* < 0.05 versus OVX + E_2_, *n* = 8–10. OVX: ovariectomy; RAL: raloxifene (1 mg/kg/day, gavage); E_2_ (50 μg/kg, s.c.); CMC: carboxymethylcellulose (1 ml/kg/day, gavage)

The increase in adipocyte size is closely associated with local adipose tissue hypoxia, contributing to the induction of HIF-1α and subsequent inflammatory responses [24]. Consistent with the histological findings of adipocyte size, protein expression of HIF-1α was markedly increased in OVX and OVX + CMC groups when compared with the Sham group, whereas significant reductions were observed in OVX + E_2_ and OVX + RAL groups when compared with OVX and OVX + CMC groups (Fig. 4a) (*P* < 0.05). Moreover, protein expression of VEGF-A, the HIF-1α downstream transcriptional target, was significantly induced in the OVX and OVX + CMC groups as compared with the Sham group (*P* < 0.05). Interestingly, this change of VEGF-A expression by OVX was dramatically attenuated by RAL treatment, but not by E_2_ replacement (Fig. 4b).

As HIF-1α induction is reported to be accompanied with inflammatory responses [25], we examined proinflammatory adipokines MCP-1 and TNF-α proteins, as well as transcription factor NF-κB p65 expression. As expected, MCP-1, TNF-α and p65 proteins were significantly elevated in OVX and OVX + CMC groups as compared to the Sham group. Administration of RAL significantly prevented the increase of MCP-1, TNF-α and p65 proteins. However, E_2_ did not show inhibitory effects on these inflammatory proteins (Fig. 4c, d and e).

Furthermore, JNK pathway is activated by TNF-α stimulation and has been regarded as a crucial mediator of obesity and insulin resistance [26]. Expression of p-JNK was significantly higher in the OVX and OVX + CMC groups than that of Sham group. Both replacement of E_2_ and treatment with RAL drastically reduced the p-JNK levels as compared with the untreated OVX group (Fig. 4f).

### Effects of RAL on non-canonical SFRP5 and Wnt5a expression in OVX rats

**Fig. 5:**
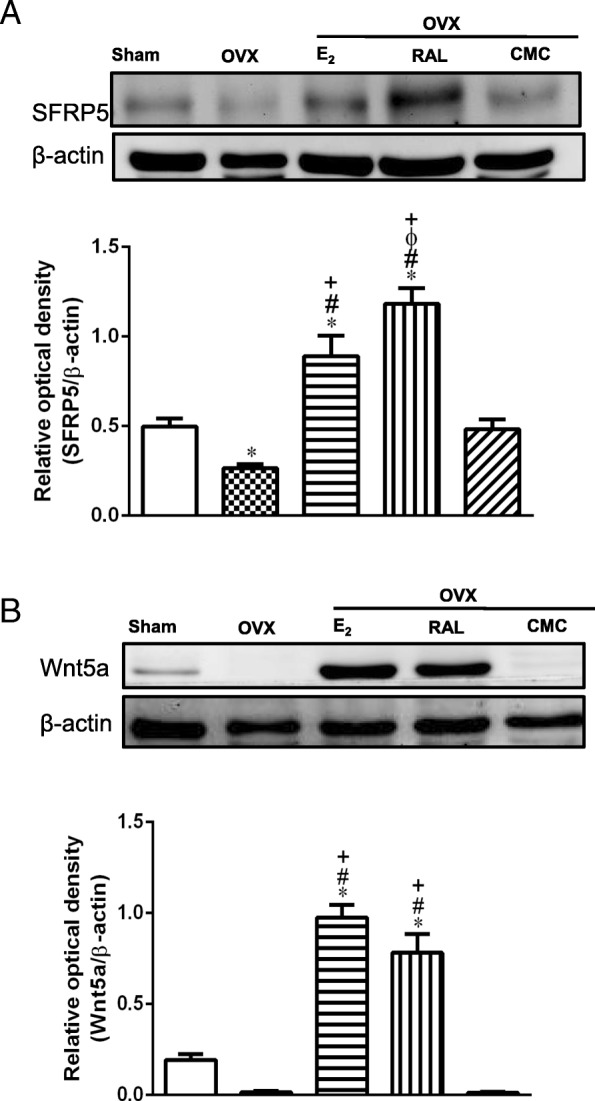
Effects of l long-term treatment with raloxifene on the expression of (**a**) SFRP5 and (**b**) Wnt5a in the retroperitoneal white adipose tissue of ovariectomized rats. Data are expressed as mean ± SEM. *P < 0.05 versus Sham; ^#^ *P* < 0.05 versus OVX; ^+^*P* < 0.05 versus OVX + CMC; ^ϕ^*P* < 0.05 versus OVX + E_2_, *n* = 8–10. OVX: ovariectomy; RAL: raloxifene (1 mg/kg/day, gavage); E_2_ (50 μg/kg, s.c.); CMC: carboxymethylcellulose (1 ml/kg/day, gavage)

Figure 5a showed that the OVX group exhibited significant lower levels of SFRP5 than those of the Sham group. Compared with the OVX group, SFRP5 expression was significantly increased in the OVX + E_2_ and OVX + RAL groups (*P* < 0.05). Similarly, E_2_ and RAL administration significantly upregulated Wnt5a expression as compared to OVX and OVX + CMC group (Fig. 5b).

### Effects of RAL on adipogenic differentiation and canonical Wnt signaling in OVX rats

**Fig. 6:**
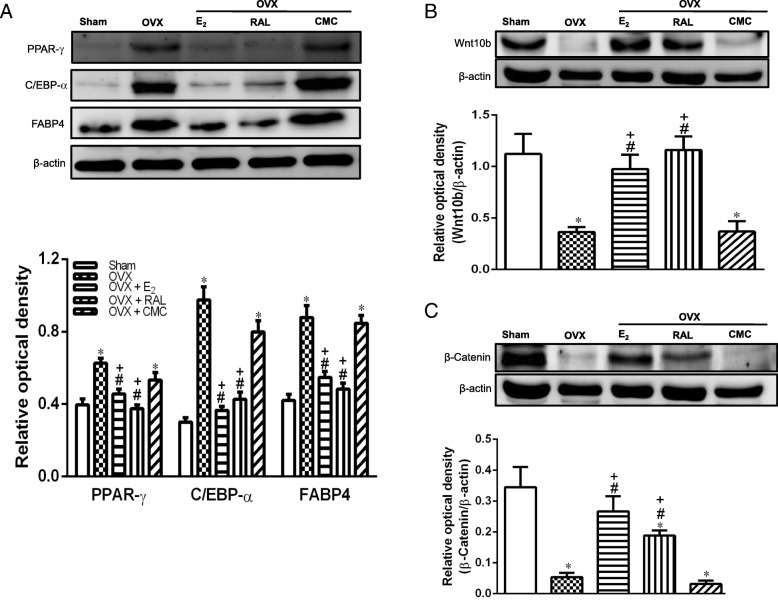
Effects of long-term treatment with raloxifene on the expression of (**a**) adipogenic proteins PPAR-γ, C/EBP-α and FABP4, and (**b**) canonical Wnt10b and (**c**) β-catenin in the retroperitoneal white adipose tissue of ovariectomized rats. Data are expressed as mean ± SEM. *P < 0.05 versus Sham; ^#^ *P* < 0.05 versus OVX; ^+^*P* < 0.05 versus OVX + CMC, n = 8–10. OVX: ovariectomy; RAL: raloxifene (1 mg/kg/day, gavage); E_2_ (50 μg/kg, s.c.); CMC: carboxymethylcellulose (1 ml/kg/day, gavage)

To examine the role RAL in the regulation of adipogenesis, we evaluated the adipogenic transcription factors such as PPAR-γ, and C/EBP-α, as well as fatty acid binding protein 4 (FABP4), an essential protein for mature adipocyte formation. As shown in Fig. 6a, the protein expression of PPAR-γ, C/EBP-α and FABP4 were significantly induced in OVX and OVX + CMC groups, which were all markedly diminished by administration with E_2_ and RAL (*P* < 0.05).

In addition, significant reduction of canonical Wnt10b and β-catenin expression was present in OVX and OVX + CMC groups and administration with E_2_ and RAL upregulated Wnt10b and β-catenin expression (Fig. 6b and c).

### Effects of RAL on lipid accumulation and adipogenesis in 3 T3-L1 cells

**Fig. 7:**
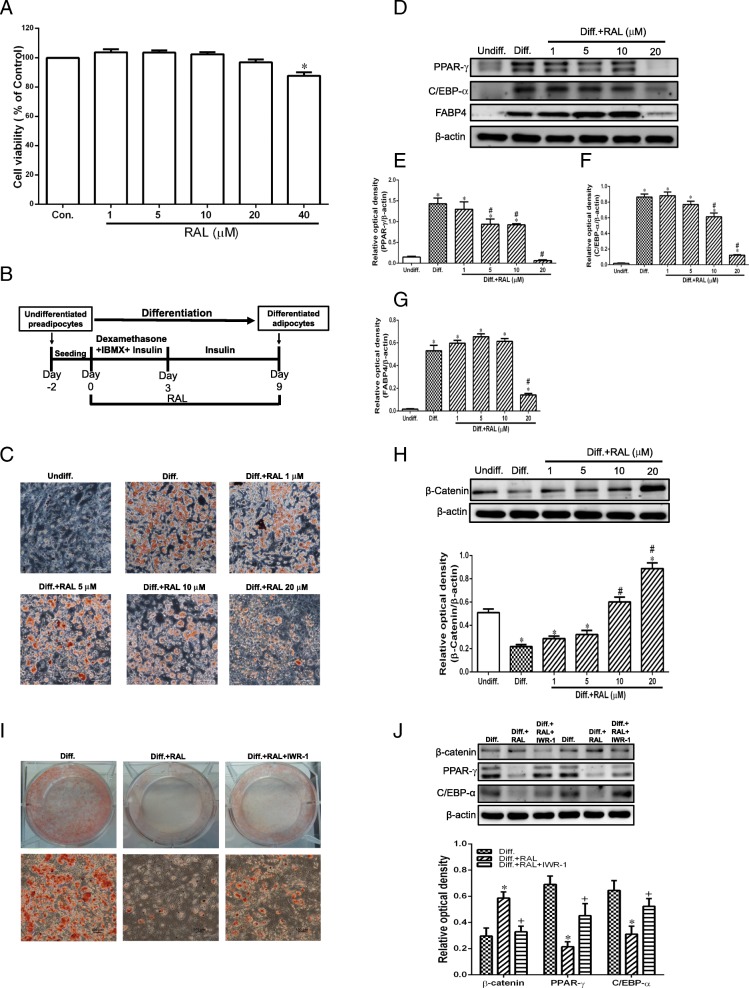
Effects of raloxifene on cell viability, lipid accumulation, adipogenic proteins and β-catenin expression in 3 T3-L1 cells. **a** cell viability of 3 T3-L1 cells treated with RAL (1–40 μM) for 16 h expressed as optical density percentage, *P < 0.05 versus control; (**b**) schematic diagram for the experimental protocol of 3 T3-L1 preadipocyte differentiation and RAL treatment; (**c**) lipid accumulation in differentiated 3 T3-L1 adipocytes stained by Oil Red O; (**d**-**g**) Western blot analysis of adipogenic proteins PPAR-γ, C/EBP-α, and FABP4 and (H) β-catenin; (**i**) effects of co-treatment with RAL (20 μM) and β-catenin inhibitor IWR-1-endo (25 μM) on oil-red-O staining and (**j**) protein expression of PPAR-γ and C/EBP-α in 3 T3-L1 adipocytes. Data are expressed as mean ± SEM. *P < 0.05 versus undifferentiation (Undiff.); ^#^ *P* < 0.05 versus differentiation (Diff.); ^*+*^*P* < 0.05 versus differentiation+ raloxifene (Diff. + RAL)

Results of cell viability assay indicated that no significant cytotoxicity was observed at various concentration of 1–20 μM of RAL treatment in 3 T3-L1 cells (Fig. 7a). Thus, these concentrations of RAL on lipid droplet accumulation were assessed using Oil Red O stain. As shown in Fig. 7c, the lipid droplet accumulation in the differentiation (diff.) group was significantly increased as compared with the undifferentiated (undiff.) group. RAL (20 μM) treatment markedly decreased the number of lipid droplets and protein expression of adipogenic markers (PPAR-γ, C/EBP-α and FABP4) (Fig. 7d-g). β-catenin expression was significantly lower in diff. Group than that of undiff. Group and administration of RAL (20 μM) markedly upregulated the expression of β-catenin (Fig. 7h). In addition, the decreases of lipid droplets and adipogenic markers PPAR-γ and C/EBP-α induced by RAL were effectively reversed by β-catenin signaling inhibitor IWR-1-endo (25 μM, Cayman Chemical, Ann Arbor, MI) (Fig. 7i and j). These results suggested that β-catenin plays an important role in mediating the anti-adipogenic effects of RAL.

### SFRP5 silencing blunted the suppressive effects of RAL on LPS-induced inflammation in 3 T3-L1 cells

**Fig. 8:**
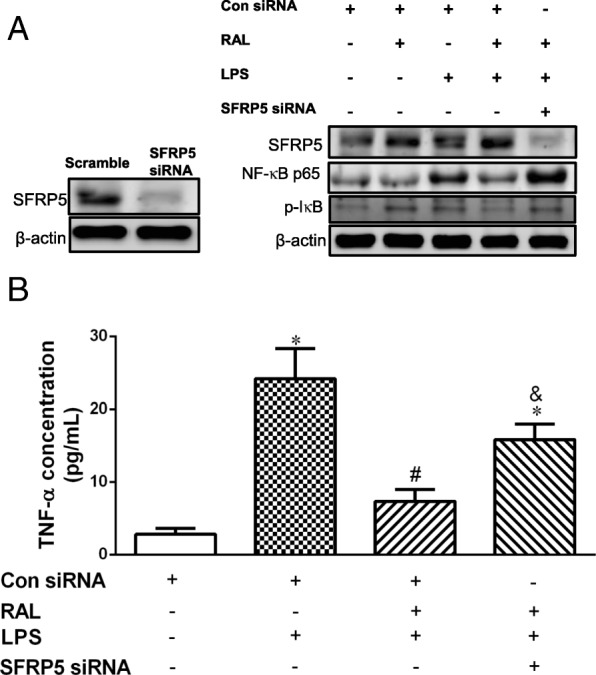
Effects of SFRP5 knockdown on the regulation of raloxifene in LPS-treated 3 T3-L1 adipocytes. **a** Western blot analysis of SFRP5, NF-κB p65 and phosphorylated I-κB protein expression in SFRP5 siRNA transfected 3 T3-L1 adipocytes treated with LPS (10 μg/mL) and RAL (20 μM). **b** Culture medium was collected to measure the TNF-α levels. Con siRNA: scramble siRNA as control; Data are expressed as mean ± SEM. *P < 0.05 versus con siRNA alone; ^#^*P* < 0.05 versus LPS + con siRNA; ^&^*P* < 0.05 versus LPS + RAL + con siRNA

As shown in Fig. 8a, LPS significantly increased NF-κB p65 and phosphorylated I-κB expression in differentiated 3 T3-L1 cells, and RAL (20 μM) suppressed the LPS-induced NF-κB p65 and phosphorylated I-κB expression, which abrogated by siRNA for SFRP5. In culture media, RAL significantly reduced the LPS-induced increases in TNF-α secretion (Fig. 8b), which also abrogated by SFRP5 siRNA.

## Discussion

The drastic increase in the prevalence of postmenopausal obesity has made recent research to pay more efforts on the pathophysiology of adipose tissue. In this study, the increase of body weight and glucose intolerance as well as adipocyte hypertrophy were ameliorated by long-term RAL treatment, revealing the importance of SERM in the regulation of metabolic changes. Our results also showed that RAL decreased HIF-1α and VEGF-A as well as proinflammatory cytokines through inhibition of NFκB and JNK pathway. This anti-inflammatory capacity might act through upregulation of SFRP5. In addition, RAL exerted anti-adipogenic effects in WAT of OVX rats and 3 T3-L1 cells via activation of canonical Wnt10b and β-catenin signaling. Thus, distinct activation of the canonical and non-canonical Wnt signaling may contribute the anti-obesity potential of RAL. These findings suggested that RAL might be considered as a therapeutic strategy for the management of postmenopausal obesity.

Evidence from both human and laboratory rodent studies has indicated that estrogen regulates the amount and distribution of WAT. Pre-menopause female exhibits lower peripheral adiposity as compared to the male profile of visceral fat accumulation with obesity, whereas this gender difference disappears after menopause [27]. Postmenopausal status accompanied with elevated testosterone and reduced estrogen levels showed a preferential increase in abdominal visceral fat evaluated by computed tomography and magnetic resonance imaging analysis [28]. In addition, a significant increase in visceral WAT was also observed in estrogen receptor α (ERα) knockout mice, suggesting estrogen is crucial for regulation of WAT deposition [29]. Previous report has demonstrated that RAL treatment shifted fat redistribution from android (abdominal region) to gynoid (hips and thighs) and reduced visceral adiposity in postmenopausal women [30]. This effect might result from the ER agonistic action of RAL in adipose tissue.

It is well known that the imbalance of food intake and energy expenditure is the major cause of obesity. Estrogen deficiency disrupts energy balance and increases tendency of body weight gain as well as fat mass [31]. We found that RAL and E_2_ treatment decreased food intake (Additional file 1: Figure S1) and might in part contribute to the preventive effect of weight gain, which is in accordance with previous study [21] and indicate that RAL decreased appetite and body weight through the regulation of leptin. On the other hand, accumulating evidence has prompted that adipocytes hyperplasia and hypertrophy are associated with the progression of obesity [32]. Thus, targeting adipogenesis and adipocyte size appear to be a potential therapeutic approach for obesity management. Our data provided important insights that RAL reduced adipocyte hypertrophy (Fig. 3) as well as suppressed adipogenesis in vivo (Fig. 6). Furthermore, the anti-adipogenic effects of RAL on cultured 3T3L-1 adipocytes were demonstrated in Fig. 7, indicating that the RAL can directly affect adipocytes to attenuate lipid accumulation, which might be mediated through activation of the Wnt-β-catenin pathway.

Impaired adipogenic differentiation during obesity promotes adipocyte hypertrophy and triggers hypoxia responses which result in the induction of the key hypoxia transcription factor HIF1-α [33]. HIF-1α is known as an important mediator in response to hypoxia that induces inflammation and angiogenesis. Under normoxia, HIF-1α is hydroxylated by prolylhydroxylase (PHD) and ubiquitinated by the E3 ubiquitin ligase via the proteasome pathway. Conversely, hydroxylation is inhibited under hypoxia and results in stabilization of the α subunit, leading to activation of HIF-1α target gene, VEGF-A and secretion of chemokines, including MCP-1 [7], TNF-α and interleukin-6 (IL-6) [8]. Disruption of HIF-1α function in adipose tissue improves high fat diet–induced obesity and insulin resistance [34]. VEGF-A is the key regulator of angiogenesis and implicated in normal and pathological vessel formation. In human obese subjects, serum VEGF is positively associated with visceral fat accumulation [35]. In addition, expression of HIF-1α and its target gene VEGF-A may also result in oxidative stress-induced NF-κB activation and subsequent up-regulation of MCP-1 and TNF-α [24, 36]. NF-κB is a transcription factor that induces the expression of various genes involved in secretion of inflammatory adipokines, which stimulates macrophage infiltration in the WAT and are involved in the development of insulin resistance. MCP-1 is a potent chemoattractant and plays an important role in the recruitment of monocytes/macrophages into the adipose tissue. MCP-1 knockout mice fed with high-fat diet exhibited lower macrophage infiltration and inflammatory responses, accompanied with improved insulin resistance and hepatic steatosis [7]. Similar studies also revealed that RAL inhibited the circulating levels of MCP-1 in postmenopausal women [37] and MCP-1 expression in human coronary artery endothelial cells [38]. Both E_2_ and RAL inhibited the MCP-1-induced monocyte migration through nongenomic ERα [39]. Thus, long-term treatment with RAL prevented estrogen deficiency-induced weight gain, visceral adiposity and metabolic abnormalities, leading to inhibition of inflammatory mediators MCP-1 and TNF-α release. Furthermore, the plasma levels of adiponectin after RAL treatment were significantly higher than that of the OVX group (Fig. 2a), suggesting that the anti-inflammatory capacity of RAL is mediated by adiponectin to inhibit the secretion of MCP-1 and improve insulin sensitivity. However, the anti-inflammatory effect of RAL in adipose tissue of OVX rats is superior to that of E_2_ (Fig. 4d & e). It may be accomplished by estrogen receptor-independent mechanisms. In a previous in vitro study of low-density lipoprotein in postmenopausal women, RAL is demonstrated to be a better antioxidant than E_2_ [40].

SFRP5 consists cysteine-rich domains and negatively regulates Wnt5a signaling by neutralizing ligands in the extracellular space [41]. Circulating SFRP5 levels are lower in both impaired glucose tolerance and type 2 diabetes patients than those of normoglycemic subjects [17]. Similar report also indicated that SFRP5 expression was reduced in rodents with genetic or dietary obesity, while systemic administration of SFRP5 improved glucose tolerance and insulin resistance through inhibition of inflammatory responses and Wnt5a-JNK signaling [18]. Thus, SFRP5-Wnt5a-JNK regulatory axis in adipose tissue serves as a potential target for the regulation of obesity-associated disorders in glucose homeostasis. Our data showed that RAL upregulated SFRP5 to counteract LPS- and estrogen deficiency-induced inflammatory responses. To verify the underlying molecular mechanisms of RAL, we conducted in vitro study using 3T3L1 cells and results showed that the suppressive effects of RAL on LPS-induced NF-κB activation and TNF-α secretion was blunted through knockdown of SFRP5. RAL augmented SFRP5-mediated non-canonical Wnt signaling to improve visceral adipose tissue dysfunction and associated metabolic impairment in OVX rats. These in vivo and in vitro data confirmed that the protective effect of RAL against adipocyte inflammation is mediated by SFRP5, which may contribute to the amelioration of obesity-associated metabolic dysfunctions.

In addition to adipocyte hypertrophy, another major characteristic of obesity-induced adipocyte remodeling is hyperplasia, which means to increase adipocyte numbers [42]. Adipocyte hyperplasia is attributed to a complicated interaction between proliferation and differentiation in preadipocytes. The key transcription factors including PPAR-γ, C/EBP-α and FABP4 were reported to play an important role in the implication of adipogenesis. PPAR-γ is a nuclear receptor in regulating development of adipose tissue, which involves genes responsible for maturation of adipocyte phenotype [43]. It also cooperates with C/EBP-α to regulate adipocyte differentiation and induces the expressions fatty acid synthase and FABP4, which is involved in the uptake, synthesis and transport of the lipids, leading to intracellular fat accumulation [44]. Intriguingly, administration of RAL significantly inhibited PPAR-γ, C/EBP-α and FABP4 production, thereby decreasing the visceral fat mass contents. Adipogenic differentiation is tightly regulated by the canonical Wnt signaling. It has been proposed that transgenic overexpression of Wnt10b in adipose tissue reduces epididymal and peri-renal fat depots by β-catenin stabilization and improves glucose intolerance [45]. In this study, RAL increased Wnt10b and β-catenin accompanied with the relatively lower levels of adipogenic markers C/EBP-α, PPAR-γ and FABP4 expressions. This anti-adipogenic effect was restored by IWR-1-endo, a specific β-catenin inhibitor that stabilizes axin-2, a scaffolding protein of the β-catenin destruction complex [46]. These findings suggest β-catenin plays a critical role in mediating RAL-induced inhibition of adipogenesis. Besides canonical Wnt, non-canonical Wnt5a also exhibits anti-adipogenic functions and undergoes osteogenesis during mesenchymal stem cell differentiation stage [47]. Based on these data, increases of canonical Wnt10b, β-catenin and non-canonical Wnt5a by RAL are strongly associated with the suppression of obesity during estrogen-deficiency.

Regarding the energy expenditure, white adipose tissue (WAT) browning plays an important role in dissipating stored energy in the form of heat [48]. It is reported that tamoxifen, the first generation SERM has been shown to possess browning effect of subcutaneous WAT [49]. It upregulates uncoupling protein-1 (UCP-1) expression, which indicates the conversion of white adipocyte into thermogenic beige adipocytes and increases in energy expenditure, unraveling the potentials of SERM in the management of obesity by upregulation of energy expenditure. Accordingly, we found that administration with RAL (10 μM) increased brown fat specific markers UCP-1, PR domain containing 16 (PRDM16), peroxisome proliferator-activated receptor gamma coactivator 1-α (PGC-1α) and cell death activator (CIDEA) protein expression in differentiated 3 T3-L1 adipocytes (Additional file 1: Figure S2), suggesting that RAL exerts browning effects of adipocytes for dissipation of energy. Collectively, the anti-obesity effects of RAL are associated with the decrease in food intake, suppression of adipogenesis and increase in energy expenditure.

FBS with charcoal stripping depletes a wide range of peptides and steroid hormones in serum including estrogen, progesterone and thyroid [50]. This procedure is designed for experimental studies associated with decreased steroid hormone interference and provides the estrogen deprivation condition [51]. Results in Fig. 1a showed that the plasma level of E_2_ was not completely depleted in the Ovx rats. Thus, charcoal stripped FBS in 3 T3-L1 cell culture was not applied to explore the underlying mechanism of RAL. Though the presence of residual estrogenic compound in FBS might affect the consequences of RAL on adipogenesis and inflammation. By comparing with the differentiation (Diff.) control group, the underlying mechanism of RAL in mediating the anti-adipogenesis and anti-inflammatory capacities can be elucidated, at least partly. Further studies might be needed to determine whether the beneficial effects of RAL still occurs in charcoal stripping FBS medium.

## Conclusion

In conclusion, RAL ameliorated estrogen deficiency-induced obesity, adipogenesis, and adipose tissue hypertrophy as well as inflammation via distinct activation of canonical Wnt10b/β-catenin and non-canonical SFRP5 signaling. RAL may be a promising therapeutic strategy for the prevention of postmenopausal obesity while targeting on the treatment of osteoporosis.

## Additional file

Additional file 1: Supplemental results. (PPTX 151 kb)

## Data Availability

The datasets analyzed in the current study are available from the corresponding author on reasonable request.
