# Supplementary figures and images for: Raloxifene inhibits adipose tissue inflammation and adipogenesis through Wnt regulation in ovariectomized rats and 3 T3-L1 cells
Source: J Biomed Sci. 2019 Aug 31;26:62. doi: 10.1186/s12929-019-0556-3 (PMC6717377; doi:10.1186/s12929-019-0556-3)

## Slide 1
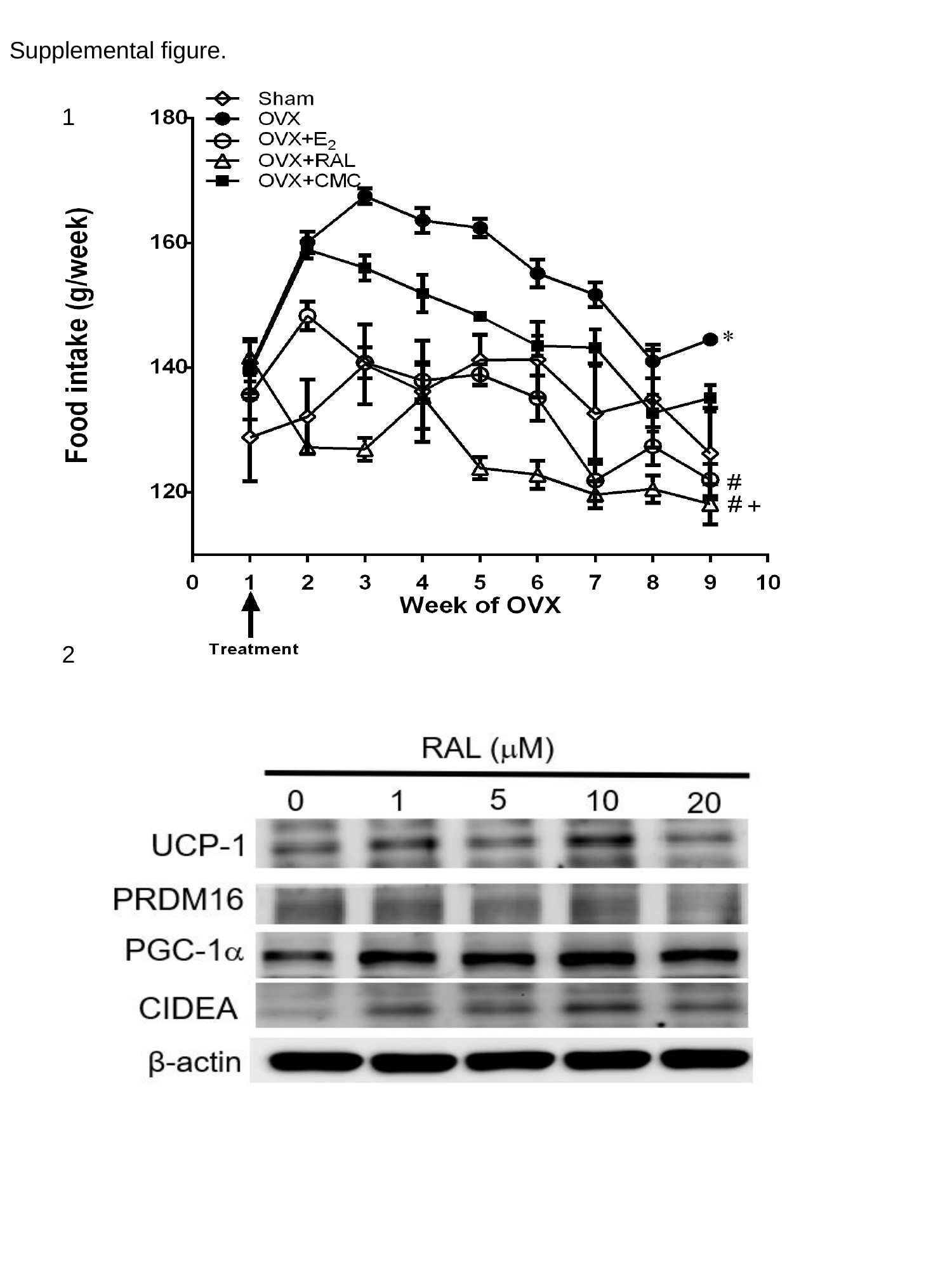

Supplemental figure.
1
2

Supplement: Supplementary file 1 — Supplemental results. (PPTX 151 kb) [file 12929_2019_556_MOESM1_ESM.pptx]
